# Spontaneous remission of congenital acute megakaryoblastic leukemia in a neonate with down syndrome

**DOI:** 10.1515/med-2025-1367

**Published:** 2026-02-25

**Authors:** Wei Yang, Sumin Sui, Sheng Hao

**Affiliations:** Department of Pediatric, The People’s Hospital of Bozhou, Bozhou, China; Department of General Pediatric, Bozhou Children’s Hospital, Bozhou, China; Department of Nephrology, Rheumatology and Immunology, Shanghai Children’s Hospital, School of Medicine, Shanghai Jiao Tong University, Shanghai, China

**Keywords:** congenital acute megakaryoblastic leukemia, spontaneous remission, down syndrome

## Abstract

**Objectives:**

Congenital acute megakaryoblastic leukemia (AMKL) is an extremely rare and aggressive malignancy with poor prognosis.

**Case presentation:**

We report a female neonate with Down syndrome (DS) who presented with respiratory distress and hepatomegaly at birth. Laboratory evaluation revealed hyperleukocytosis (253.45 × 10^9^/L) and bone marrow studies confirmed AMKL with trisomy 21. Remarkably, spontaneous hematologic remission occurred within 2 months without chemotherapy.

**Conclusions:**

This case highlights the unique clinical course of DS-associated AMKL and underscores the potential for spontaneous regression in congenital leukemia.

## Introduction

Congenital leukemia, defined as leukemia presenting in the neonatal period (0–28 days postnatally), is an exceedingly rare hematologic malignancy, with an incidence of approximately 1–5 cases per 1 million live births according to population-based studies [1]. This condition accounts for less than 1 % of all pediatric leukemia diagnoses, underscoring its rarity among perinatal oncologic disorders [2].

Acute megakaryoblastic leukemia (AMKL; AML-M7) accounts for 4–15 % of pediatric acute myeloid leukemia cases, with a predilection for infants and children under the age of two [3]. The profound link between DS and leukemia, particularly AMKL, is rooted in its unique biology. Trisomy 21 creates a pre-leukemic state, largely due to mutations in the GATA1 gene, which lead to the production of a truncated protein (GATA1s). This, combined with the gene dosage effect of chromosome 21 (e.g., overexpression of oncogenes like RUNX1 and ERG), disrupts normal megakaryocytic development and predisposes to transient abnormal myelopoiesis (TAM) and its progression to ML-DS. Congenital AMKL is exceptionally rare, with most cases associated with Down syndrome (DS). Despite its aggressive nature, spontaneous remission has been documented in rare instances, particularly in DS-related cases. We present a neonate with DS-associated AMKL who achieved spontaneous remission without cytotoxic therapy.

## Case presentation

**Figure 1:**
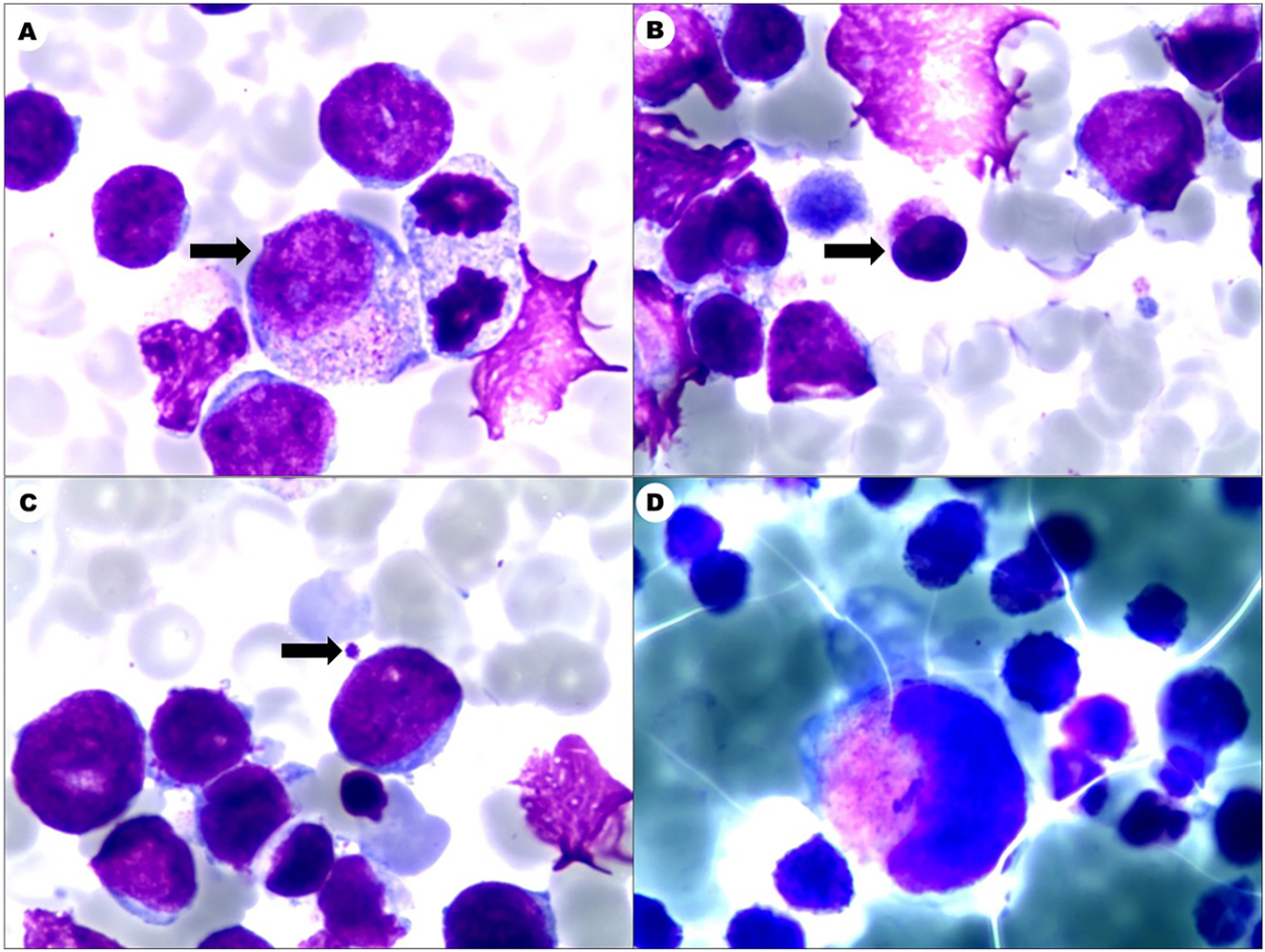
Marrow demonstrated acute myeloid leukemia (AML), bone marrow hyperplasia was significantly active, with primitive cells accounting for 72.5 %. (A) Promyelocyte (black arrow), (B) lymphoid megakaryocyte (black arrow), (C) platelet (black arrow), (D) granular megakaryocyte (A–D Leishman stain, 1,000×).

**Figure 2:**
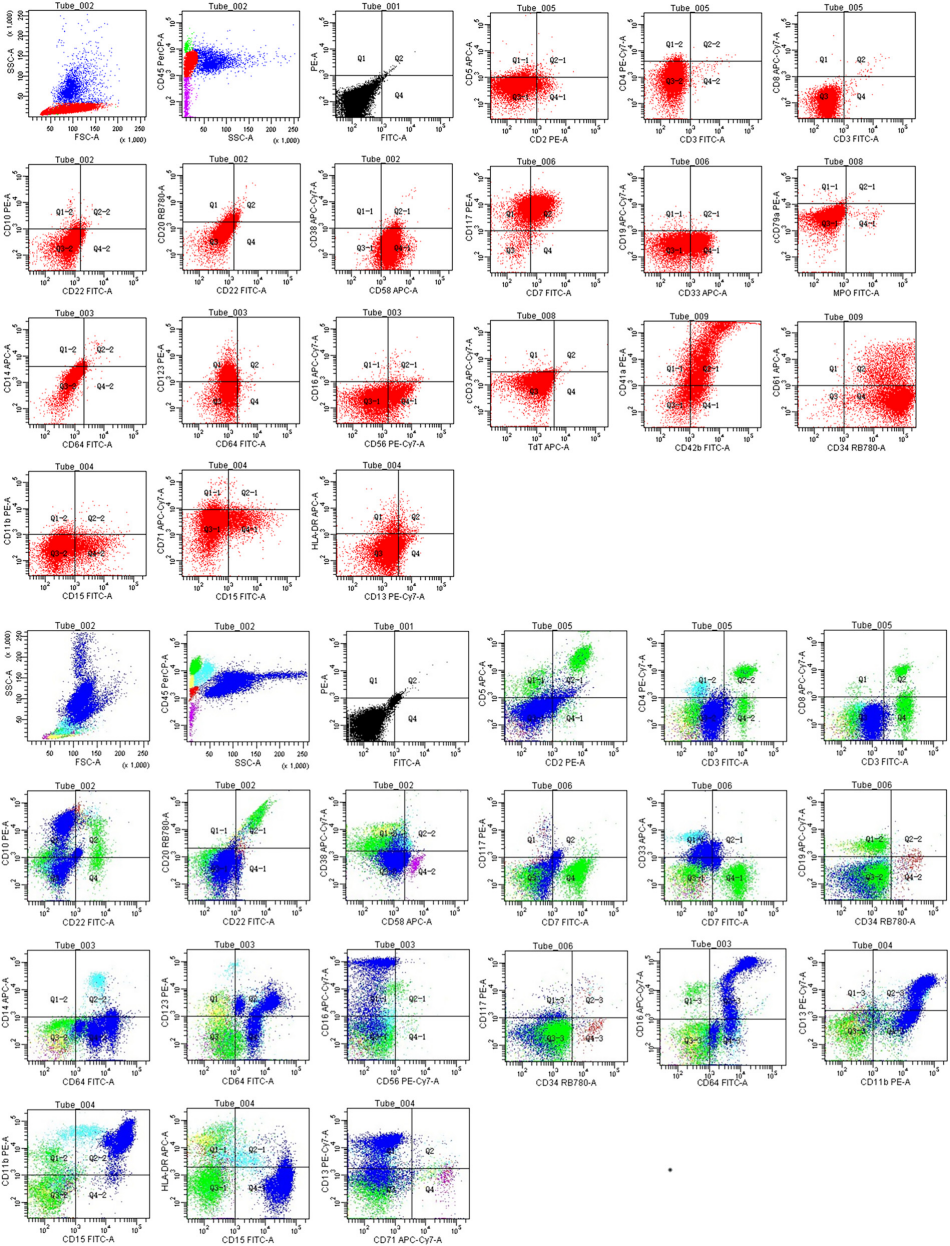
Comparative flow cytometric immunophenotyping of bone marrow. A. Patient sample. Analysis reveals a dominant abnormal blast population (77 % of nucleated cells) expressing CD7, CD33, CD34, CD41a, CD42b, CD56, CD58, CD61, CD117, and CD123, with subset CD15 positivity. The immunophenotype, particularly the co-expression of CD41a, CD42b, and CD61, is diagnostic of acute megakaryoblastic leukemia (AML-M7). Lymphoid precursors are significantly reduced. B. Normal control. Gating on CD45/SSC shows a physiological distribution: lymphocytes (34 %), a small blast population (2 %), monocytes (5.5 %), and granulocytes (52.5 %) without phenotypic abnormalities. The CD41a/CD61 plot shows a distinct, small population of mature megakaryocytes, starkly contrasting with the aberrant blast pattern in (A).

A female infant was delivered via cesarean section at 38^4^/_7_ weeks due to fetal distress, with Apgar scores of 8 (1 min) and 9 (5 min). The neonate exhibited respiratory distress (RR 66 bpm, SpO_2_ 84 %), grunting, and cyanosis. Physical examination revealed hepatomegaly (liver 4 cm below the costal margin) and generalized meconium staining. Abdominal ultrasound confirmed hepatomegaly with diffusely heterogeneous echotexture, though no definitive signs of fibrosis were noted. Initial laboratory evaluation revealed striking hyperleukocytosis (253.45 × 10^9^/L) with monocytic predominance (73.4 %), mild anemia (Hb 104 g/L), orthoplastocyte (156 × 10^9^/L), metabolic acidosis (HCO_3_^−^ 9.8 mmol/L), hyperkalemia (8.28 mmol/L), and elevated cardiac enzymes (CK-MB 203 U/L, LDH 4613 U/L). Bone marrow analysis demonstrated acute myeloid leukemia (Figure 1) with hypercellular marrow (72.5 % blasts, POX−, PAS focal+). Flow cytometry was performed on a BD FACS Canto II instrument using BD FACSDiva software. The analysis utilized antibodies against CD34 (clone 8G12), CD117 (104D2), CD33 (WM53), CD7 (M-T701), CD56 (NCAM16.2), CD41a (HIP8), CD42b (HIP1), and CD61 (VI-PL2) (all from BD Biosciences). The analysis showed an abnormal blast population (77 % of nucleated cells) expressing CD34, CD117, CD33, CD7, CD56, CD123, and megakaryocytic markers (CD41a/CD42b/CD61), with partial CD15 (Figure 2), confirming the diagnosis of AML-M7. Bone marrow karyotype showed trisomy 21 karyotype (47, XX+21[20], Figure S1). An echocardiogram revealed patent foramen ovale and patent ductus arteriosus with left-to-right shunt. Cranial ultrasound reported subependymal hemorrhage and periventricular leukomalacia. Whole-exome sequencing (WES) was performed on DNA from bone marrow and peripheral blood. Library preparation was conducted using the IDT xGen Exome Research Panel v2, and sequencing was performed on an Illumina NovaSeq 6000 platform. Data analysis was performed using the Sentieon pipeline, with variants filtered against population databases (gnomAD) and cancer databases (COSMIC). The mean coverage depth was 100×, and the variant allele frequency threshold was set at >5 %. WES revealed no pathogenic germline or somatic mutations in known leukemia-associated genes (e.g., GATA1, FLT3, NPM1), with a mean coverage depth of 100× and variant allele frequency threshold >5 %. Parents declined chemotherapy. Supportive care included comprehensive respiratory support, intravenous albumin infusion and infection prophylaxis. Serial monitoring showed progressive hematologic improvement, with WBC reaching 10.27 × 10^9^/L, Hb 120 g/L, and platelets 417 × 10^9^/L by day 90. The patient continues to be followed up and remains in hematologic remission at 6 months of age. Initial neurodevelopmental assessments are within expected parameters for her age, considering her DS diagnosis, with no overt deficits detected to date.

Ethical approval was obtained from the Ethics Committee of Bozhou People’s Hospital. Written informed consent was obtained from the parents for both treatment and publication of this case report, including all clinical data and images. All patient identifiers have been removed to protect confidentiality.

## Discussion

DS is associated with transient abnormal myelopoiesis (DS-TAM) in newborns. Myeloid leukemia of DS (ML-DS) is characterized by megakaryoblastic phenotype, early age of onset, and favorable response to chemotherapy [4]. The diagnosis of congenital AMKL in our DS neonate, as opposed to the more common and often self-limiting DS-TAM, was based on several key factors. While DS-TAM typically presents at birth and resolves within the first few months, our patient’s presentation was severe, with extreme hyperleukocytosis (>250 × 10^9^/L) and significant hepatomegaly, features more consistent with overt leukemia. Furthermore, the persistence of blasts and requirement for supportive care beyond the typical window for TAM resolution supported the diagnosis of AMKL.

Although the absence of a GATA1 mutation is atypical, it does not preclude the diagnosis of AMKL, as the immunophenotype and clinical course were definitive. Our case of spontaneous remission in a DS neonate without detectable *GATA1* mutations presents a fascinating paradox. The absence of a *GATA1* mutation, a cornerstone of DS-associated leukemogenesis, prompts consideration of alternative mechanisms. Technical limitations, such as low-level mosaicism below the detection threshold of WES, cannot be entirely ruled out. More intriguingly, this finding may point to the existence of non-mutational drivers, such as epigenetic modifications or dysregulation of other genes on chromosome 21 (e.g., *ERG*, *RUNX1*), which could transiently disrupt megakaryopoiesis. The spontaneous clearance of the leukemic clone further suggests a potential role for the unique immune microenvironment in neonates with DS, which may be primed for effective immune surveillance and eradication of pre-malignant cells.

The enigmatic nature of this case, particularly the lack of a clear genetic driver and the phenomenon of spontaneous remission, highlights the limitations of traditional analytical methods when dealing with rare “n-of-1” scenarios. This is precisely where artificial intelligence (AI) holds immense promise for advancing the field. Recent breakthroughs in AI, such as the AlphaFold model which has revolutionized structural biology and drug discovery [5], and the impressive capabilities of large language models in diverse medical contexts [6], demonstrate the power of machine learning to identify complex patterns from large, multimodal datasets. In rare diseases, where large sample sizes are unattainable, cases like ours become invaluable. They serve as crucial “small data” points that can challenge and refine AI models. Future research directions should focus on aggregating such rare cases into international registries and employing AI to integrate multi-omics data (genomics, transcriptomics, proteomics) with detailed clinical phenotypes. The goal would be to uncover the hidden biological signatures of spontaneous remission, which could ultimately lead to the development of AI-powered clinical decision support tools. Such tools could help risk-stratify future DS neonates with hematologic abnormalities, identifying those who might benefit from a watchful waiting approach vs. those who require immediate intervention, thereby moving towards truly personalized management for these rare and complex patients.

## Learning point for clinicians

Our case underscores the importance of closely monitoring neonates with DS who present with hematologic abnormalities. While aggressive treatment is often necessary for congenital leukemia, our patient’s spontaneous remission suggests that a watchful waiting approach may be considered in select cases, especially when the clinical course is relatively stable.

## Supplementary Material

Supplementary Material

Supplementary Material
