# Supplementary figures and images for: Spontaneous remission of congenital acute megakaryoblastic leukemia in a neonate with down syndrome
Source: Open Med (Wars). 2026 Feb 25;21(1):20251367. doi: 10.1515/med-2025-1367 (PMC12949609; doi:10.1515/med-2025-1367)

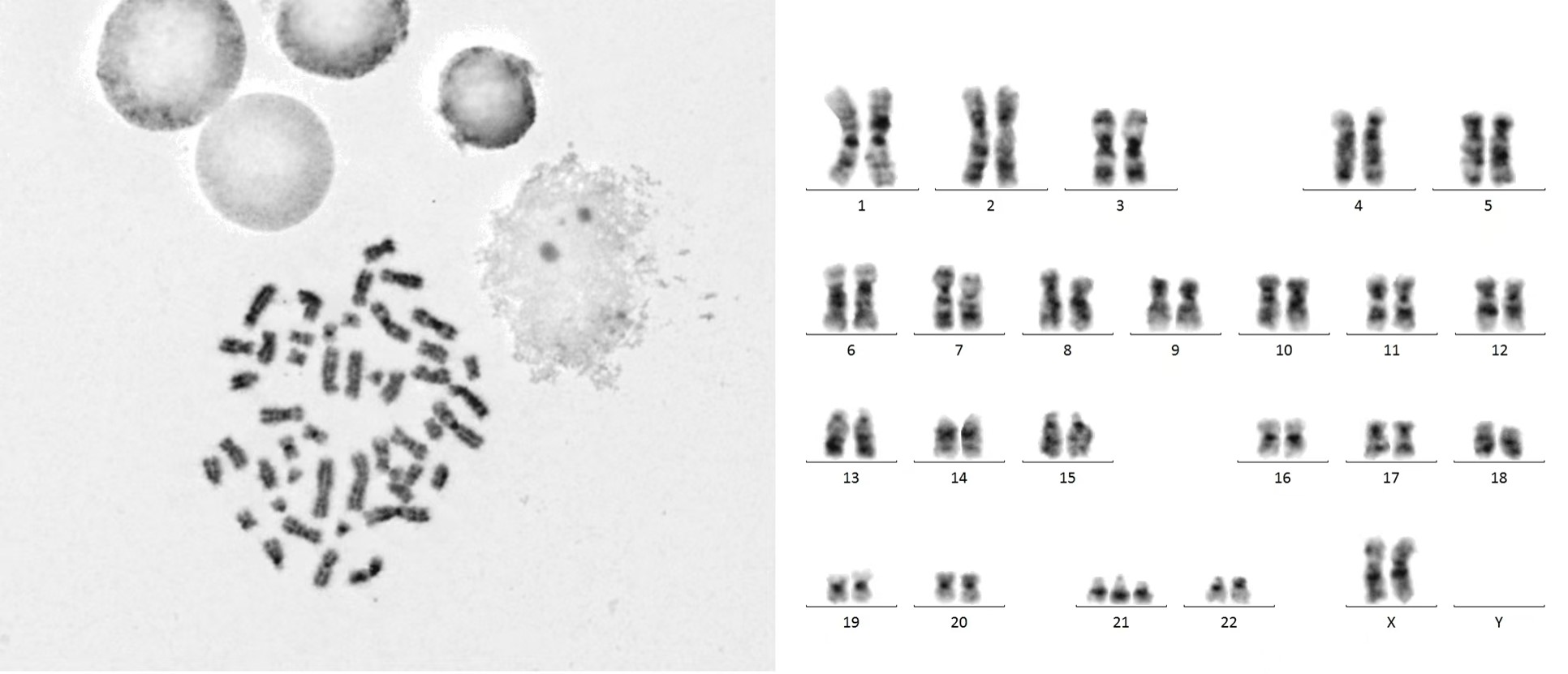

Supplement: Supplementary file 1 — Supplementary Material [file j_med-2025-1367_suppl_001.jpg]
