# Supplementary material for: Spontaneous remission of congenital acute megakaryoblastic leukemia in a neonate with down syndrome
Source: Open Med (Wars). 2026 Feb 25;21(1):20251367. doi: 10.1515/med-2025-1367 (PMC12949609; doi:10.1515/med-2025-1367)
Supplement: Supplementary file 2 — Supplementary Material [file j_med-2025-1367_suppl_002.docx]

Figure S1: Karyotype Analysis of bone marrow (Giemsa staining) demonstrated a 47, XY, +21 karyotype (male) consistent with trisomy 21. Three distinct copies of chromosome 21 are observed with characteristic G-banding patterns.
